# Extracting Total Anthocyanin from Purple Sweet Potato Using an Effective Ultrasound-Assisted Compound Enzymatic Extraction Technology

**DOI:** 10.3390/molecules27144344

**Published:** 2022-07-06

**Authors:** Fang Wang, Shuo Zhang, Guowei Deng, Kun Xu, Haiyan Xu, Jialei Liu

**Affiliations:** 1 Sichuan Provincial Key Laboratory for Development and Utilization of Characteristic Horticultural Biological Resources, College of Chemistry and Life Sciences, Chengdu Normal University, Chengdu 611130, China; wangfangbia@163.com (F.W.); 15388114168@163.com (S.Z.); 091044@cdnu.edu.cn (K.X.); 2 College of Life Sciences, Sichuan Normal University, Chengdu 610101, China; weiliangxhy@163.com; 3 Institute of Environment and Sustainable Development in Agriculture, Chinese Academy of Agricultural Sciences, Beijing 100081, China

**Keywords:** anthocyanins, ultrasound-assisted compound enzymatic extraction, purple sweet potato, response surface methodology, antioxidant activity

## Abstract

This study aimed to develop an effective technique for extracting total anthocyanins from purple sweet potato (Mianzishu 9) (PSP9) by ultrasound-assisted compound enzymatic extraction (UAEE). Single-factor experiments, Plackett-Burman experimental design, and response surface methodology were utilized for optimizing extraction conditions, and the antioxidant activities were evaluated. Anthocyanins were also measured using an ultra-performance liquid chromatograph linked to a mass spectrometer (UPLC-MS). The maximum yield of total anthocyanins was 2.27 mg/g under the following conditions: the ethanol concentration was 78%, the material-to-liquid ratio was 1:15 g/mL, the enzyme ratio (cellulase: pectinase: papain) was 2:2:1 and its hydrolysis was at 41 °C, pH = 4.5, 1.5 h, the ultrasonication was at 48 °C and conducted twice for 20 min each time. In addition to higher yield, anthocyanins extracted from purple sweet potato by UAEE showed great ability to scavenge DPPH (IC_50_ of 0.089 μg/mL) and hydroxyl radicals (IC_50_ of 100.229 μg/mL). Five anthocyanins were found in the purple sweet potato extract from UAEE. Taken together, the ultrasound-assisted compound enzymatic method can rapidly and effectively extract anthocyanins with greater antioxidant capacity from purple sweet potato.

## 1. Introduction

In the past decades, multiple artificial food colorants have been utilized in processed foods due to their high stability and low cost [1]. However, increasing scientific evidence demonstrates the toxicity of artificial food colorants, causing great concern. These toxic food colorants may be potentially carcinogenic, teratogenic and/or mutagenic, and could result in birth defects and various cancers [2]. Therefore, natural food pigments, including anthocyanins, betanin, carotenoids, and chlorophyllins, are considered safe alternatives and officially approved for utilization in foods [3].

Anthocyanins represent a group of water-soluble antioxidant flavonoids with high amounts in fruits and vegetables, conferring blue, red, and purple colors to plants [4]. Mounting evidence indicates foods with high anthocyanin levels have several health benefits through their antioxidant, anti-inflammatory, anticarcinogenic, chemoprotective, and antihyperglycemic properties, and can also prevent the oxidation of LDL-cholesterol [5]. Generally, anthocyanin-rich plants are berries, including blueberry [6], cranberry [7], strawberry [8], and sweet cherry [9]. However, the high cost of berries makes natural production of anthocyanins relatively expensive, indicating the need for identifying inexpensive natural plants with high levels of anthocyanins, as low-cost sources of anthocyanins.

Purple sweet potato is considered a healthy food due to its unique flavor, high fiber amounts and health benefits. Studies have demonstrated its potent anti-hyperglycemic and anti-hyperuricemic [10], antioxidant [11], anti-obesity [12], and anti-inflammatory [13] effects, which are likely exerted by its bioactive constituents, such as anthocyanins, phenolic acid, and alkali-soluble polysaccharide. Anthocyanins from purple sweet potato are relatively stable under multiple storage conditions, such as under various lights, different pH levels, and various temperatures, and are resistant to processing. As a result, of these properties, anthocyanins have gained high popularity. However, anthocyanin content in purple sweet potato is relatively low, with only 0.10–0.97 mg/g in fresh weight [14,15]. Developing more effective methods for the extraction of purple sweet potato anthocyanins, to be used as a natural colorant in the food industry, is urgently required. Mianzishu 9 was selected from the Mianyang Institution of Agriculture Science and Southwest University by group cross. It is a variety of purple sweet potato with a high content of anthocyanins (*Dioscorea esculenta* Lour. Burkill No. 9), having an elevated yield, a high commodity rate, good cooking quality, tolerance to storage conditions, and resistance to diseases [16]. However, there has not been, as far as we know, a comprehensive study of total anthocyanin from PSP9.

Anthocyanin extraction from plant materials by conventional solvent extraction, ultrasonic-assisted extraction [17], freeze-ultrasonic thawing technology [6], water extraction with high-pressure carbon dioxide [18], and ultrasonic-assisted enzymatic extraction [19] have been previously reported on. Ultrasonic-assisted extraction is a significant advance over previous extraction processes since it is simple to use, inexpensive, fast, and only takes a little volume of solvent. It does not require any complex maintenance procedures and has been widely used all over, with much information on its properties and methods of application [20]. Furthermore, the impact of ultrasound in the extracting medium induces cavitation, which encourages cell rupture and the conveyance of the analytes of interest within the solution. The use of enzymatic procedures, such as cellulase and pectinase, as well as ultrasonic processing for anthocyanin extraction from natural plant resources, has improved anthocyanin output [21]. The advantages of both ultrasonic and enzymatic-assisted extraction techniques are combined, resulting in efficient and ecologically friendly alternatives to standard anthocyanin extraction methods. Nonetheless, there has yet to be a report on purple sweet potato ultrasonic-assisted enzymatic extraction (Mianzishu 9). Hence, ultrasonic-assisted enzymatic extraction of anthocyanins from PSP9 and the optimal extraction conditions were here examined.

For the separation and measurement of anthocyanins in food matrixes, several techniques, such as liquid chromatography with ultraviolet/visible detection or liquid chromatography-mass spectrometry have been used (LC-UV-vis and LC-MS) [22,23]. However, no one has yet documented the separation, purification, and identification of anthocyanins from PSP9 using LC-MS.

The goal of this study was to investigate the composition of anthocyanins in PSP9 and promote a higher commercial value through the development of an anthocyanin-based coloring extract. The final method should be suitable for industrial applications as well as commercial laboratory analysis. A thorough literature search revealed no references or reports on the optimization of anthocyanin compound extraction from PSP9 to the best of our knowledge. Therefore, we optimized extraction conditions by single-factor experiments, Plackett-Burman experimental design, and response surface methodology, with anthocyanin yield as the outcome. The antioxidant ability of total anthocyanins was carried out to determine the extract’s efficacy in the UAEE technique. The primary anthocyanins were discovered and described using UPLC-MS, adding significantly to our understanding of their nutraceutical composition.

## 2. Results and Discussion

### 2.1. Single-Factor Tests of Total Anthocyanin Extraction

**Figure 1:**
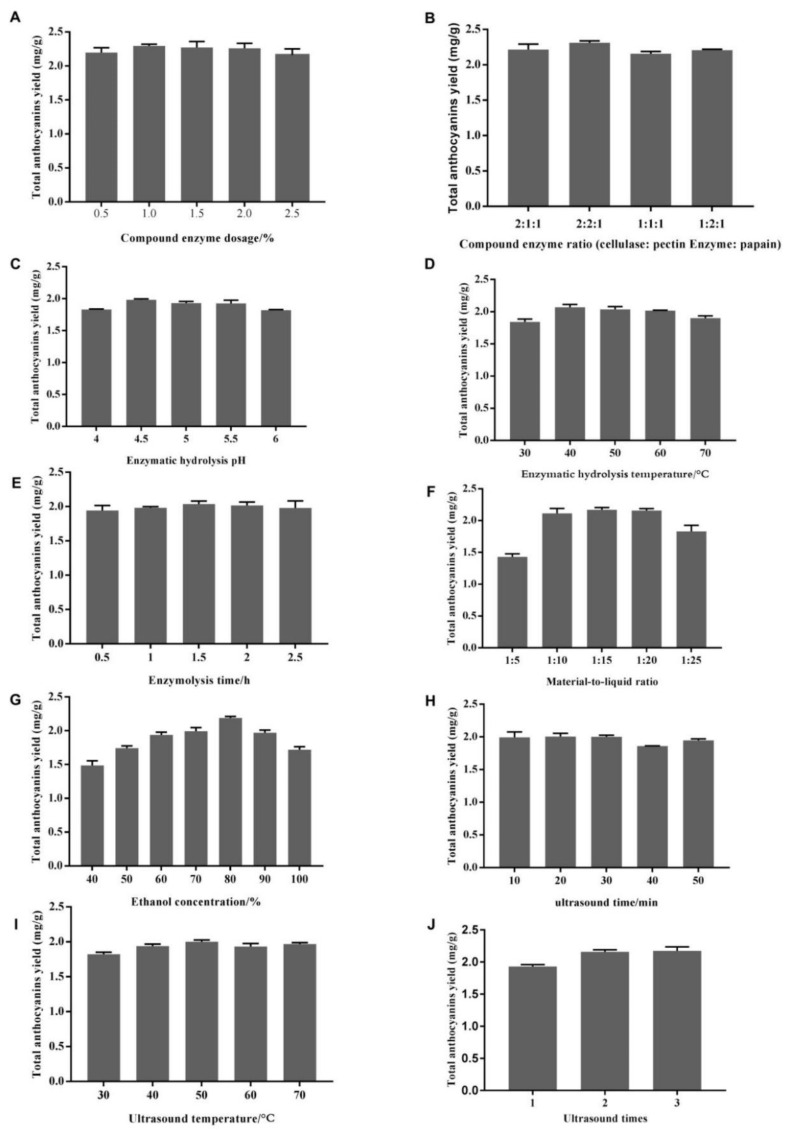
Effects of compound enzyme dosage (**A**), compound enzyme ratio (cellulase: pectinase: papain) (**B**), enzymatic hydrolysis pH (**C**), enzymatic hydrolysis temperature (**D**), enzymolysis time (**E**), material-to-liquid ratio (**F**), ethanol concentration (**G**), ultrasound time (**H**), ultrasound temperature (**I**), and number of ultrasonication sessions (**J**) on total anthocyanin extraction yield.

In single-factor analysis, ethanol concentration, material-to-liquid ratio, compound enzyme dosage, compound enzyme ratio (cellulase: pectinase: papain), enzymatic hydrolysis temperature, pH, and enzymatic hydrolysis time, ultrasound temperature, ultrasound time, and number of ultrasonication sessions were varied to obtain optimal total anthocyanin yield (Figure 1).

As shown in Figure 1, total anthocyanin yield varied under various extraction conditions. It gradually increased with compound enzyme dosage from 0.5% to 1.0% (Figure 1A), but further dosage increase resulted in lower yield. A high dosage of the compound enzyme contributed to a high degree of enzymolysis in purple sweet potato, accelerating anthocyanin dissolution. Thus, a compound enzyme dosage of 1.0% was applied in subsequent experiments.

Next, total anthocyanin yield increased with compound enzyme ratio (cellulase: pectinase: papain) (Figure 1B) up to 2.26 mg/g for 2:2:1. Cellulase promotes the dissolution of anthocyanins by destroying the cell wall of purple sweet potato, and pectinase catalyzes the interstitium of purple sweet potato to hydrolyze it into pectic and galacturonic acids [24], which results in the release of anthocyanins from cells. Papain can hydrolyze proteins in purple sweet potato to prevent the oxidation of anthocyanins, thus improving its yield [25]. Thus, the compound enzyme ratio (cellulase: pectinase: papain) was set at 2:2:1 for further experiments.

The effect of enzymatic hydrolysis pH (4, 4.5, 5, 5.5, and 6) was tested next, and extraction efficiency was maximized at pH 4.5 (Figure 1C). Since the thermal stability of anthocyanins influences their effects, various enzymatic hydrolysis temperatures, i.e., 30, 40, 50, 60, and 70 °C, were also tested. The maximum extraction yield of 2.07 mg/g was obtained at the enzymatic hydrolysis temperature of 40 °C; however, the yield decreased thereafter (Figure 1D). As for reaction time, anthocyanin yield increased progressively with enzymatic hydrolysis time and reached 2.03 mg/g at 1.5 h, but decreased with longer duration (Figure 1E).

Material-to-liquid ratio is considered a major factor in total anthocyanin extraction [9]. Extraction yield increased at ratios from 1:5 to 1:15, reaching up to 2.17 mg/g at 1:15 with no further increase (Figure 1F). A high solid-liquid ratio might enhance contact between solvent and plant materials, thereby elevating anthocyanin yield [26]. However, with a solid-liquid ratio, oxygen content in the extract increases, resulting in the oxidation of some anthocyanins, which decreases the yield of anthocyanins [27]. Taking into consideration the cost and environmental concerns, 1:15 was used for efficiently extracting anthocyanins.

According to research, ethanol is the most effective solvent for extracting anthocyanins from various materials [28]. Increasing ethanol concentration initially increased extraction yield up to 80%, followed by a decrease (Figure 1G), which may be explained by the principle of similarity compatibility [6]. In this study, 80% ethanol was utilized as the optimal extraction solvent.

The effect of ultrasound duration (10, 20, 30, 40, and 50 min) was examined, and maximum extraction efficiency was found at a duration of 30 min (Figure 1H). Since the thermal stability of anthocyanins influences their activities, various ultrasound temperatures (30, 40, 50, 60, and 70 °C) were also tested. The maximum extraction yield (2.00 mg/g) was obtained at an ultrasound temperature of 50 °C, but decreased with longer duration (Figure 1I). In addition, anthocyanin yield increased progressively with the number of ultrasonication sessions, to peak at 2.17 mg/g after 2 sessions (Figure 1J). Taking into consideration the cost and environmental concerns, 2 sessions were applied for efficient extraction.

### 2.2. Screening of Significant Variables Using the Plackett-Burman Design (PBD)

The effects of ten factors on total anthocyanin yield were investigated using the PBD. The PBD matrix used Y (total anthocyanin yield) as a response factor, and ANOVA results are depicted in Table 1. For total anthocyanin yield, compound enzyme dosage (X_1_) and ethanol concentration (X_5_) showed negative effects, whereas enzymatic hydrolysis temperature (X_3_) and ultrasound temperature (X_6_) had positive effects. On total anthocyanin yield, enzymatic hydrolysis pH (X_2_) and material-to-liquid ratio (X_4_) had non-significant effects (*p* > 0.05).

The first-order model derived from the PBD for total anthocyanin yield is as follows (Equation (1)):

Y = 1.96 − 0.054 X_1_ + 4.082E − 0.03 X_2_ + 0.028 X_3_ + 3.791E − 0.03 X_4_ − 0.19 X_5_ + 0.028 X_6_ (1)

Regarding the non-selected variables pertaining to total anthocyanin yield and extraction efficiency, 80% ethanol concentration (X_5_), 50 °C ultrasound temperature (X_6_), 1.0% compound enzyme dosage (X_1_), and 40 °C enzymatic hydrolysis temperature (X_3_) from single factor analysis were selected for optimization assays.

### 2.3. Optimization by BBD

Ethanol concentration, ultrasound temperature, compound enzyme dosage, and enzymatic hydrolysis temperature were further optimized by the BBD (arguments and results for responses in Table 2). Twenty-nine assays were performed (data in Table 2). Extraction efficiency was assessed as shown in the following polynomial equation (Equation (2)) on the basis of the regression analysis of experimentally generated data:

Y = 2.3 − 0.053 X_1_ + 0.013 X_2_ + 0.022 X_3_ + 0.002278 X_4_ − 0.033 X_1_ X_2_ − 0.084 X_1_ X_3_ + 0.02 X_1_ X_4_ − 0.09 X_2_ X_3_ + 0.048 X_2_ X_4_ + 0.061 X_3_ X_4_ − 0.2 X_1_^2^ − 0.056 X_2_^2^
− 0.06 X_3_^2^ − 0.14 X_4_^2^ (2)

where Y is total anthocyanin yield; X_1_, X_2_, X_3_ and X_4_ are coded variables for ethanol concentration, ultrasound temperature, compound enzyme dosage and enzymatic hydrolysis temperature, respectively.

Table 3 depicts ANOVA results for the response surface model of the four factors, providing evidence for a logical prediction of total anthocyanin yield by the probability (*p* < 0.0001) and F-values (16.93). The lack of fit test was performed to assess whether the experimental and theoretical models were consistent. The lack of fit result was not statistically different from the pure error (*p* value for total anthocyanin yield, 0.2964; F-value for both responses, 1.82). Therefore, there was an agreement between model and experimental values, accurately predicting total anthocyanin yield.

Generally, an elevated coefficient of determination (R^2^) suggests the model yields a credible fit for the data. Here, R^2^ for total anthocyanin yield (0.9442) was above 0.98 and in agreement with adjusted R^2^ (0.8884 for total anthocyanin yield), indicating a good fit; thus, the model was considered to be reliable for predictions. The coefficient of variation (CV) is frequently utilized to assess the local variability of statistical data; a low CV usually suggests the appropriateness of a model to fit the measured values. The CV for total anthocyanin yield was 2.07%, which is acceptable for expressing results adequately.

The coefficients obtained for X_1_ were significant (*p* < 0.01), indicating ethanol concentration significantly affects the rate of anthocyanin extraction. By contrast, the coefficients determined for X_2_, X_3_, and X_4_ were not significant, suggesting that the effects of ultrasound temperature, compound enzyme dosage, and enzymatic hydrolysis temperature on extraction rate were not significant (*p* > 0.01). The quadratic terms X_1_^2^, X_2_^2^, X_3_^2^ and X_4_^2^, as well as the interactive terms X_1_ X_3_, X_2_ X_3_ and X_3_ X_4_, showed statistical significance (*p* < 0.05), while the remaining coefficients had no statistical significance (*p* > 0.05). The above findings reveal an overt quadratic association of these three parameters with the rate of anthocyanin extraction. The “numerical analysis” in the “optimization program” software suggested that factor conditions yielding maximum response could be identified. The optimal conditions for total anthocyanin extraction from PSP9 included ethanol concentration (77.1%), ultrasound temperature (47.70 °C), compound enzyme dosage (1.28%) and enzymatic hydrolysis temperature (40.73 °C). The predicted yield for total anthocyanin extraction under such conditions was 2.31 mg/g.

### 2.4. Analysis Contour and Response Surface

**Figure 2:**
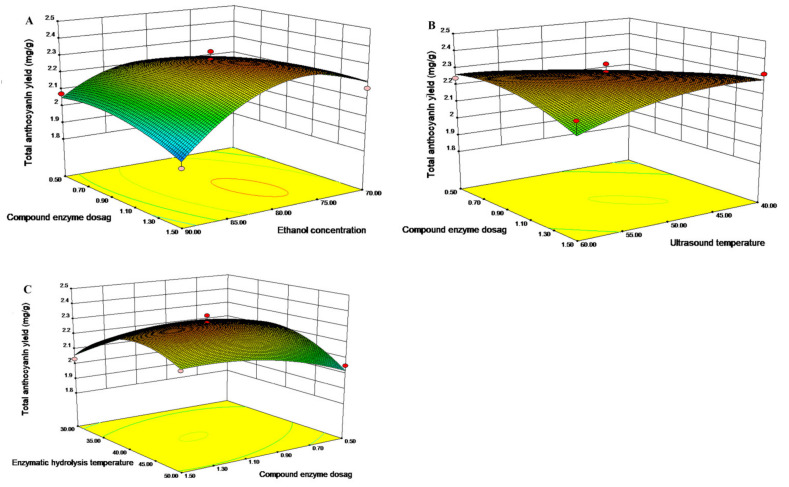
Response surface plots for determining interactions among three independent extraction factors affecting total anthocyanin extraction yield. (**A**). Total anthocyanin content versus ethanol concentration and compound enzyme dosage. (**B**). Total anthocyanin content versus ultrasound temperature and compound enzyme dosage. (**C**). Total anthocyanin content versus compound enzyme dosage and enzymatic hydrolysis temperature.

The impacts of factors and interactions on total anthocyanin yield were assessed based on three-dimensional response surfaces, and plotted by response that was dependent on the two variables (Figure 2). The interaction of ethanol concentration and compound enzyme dosage for total anthocyanin yield was examined (Figure 2A), while ultrasound temperature and enzymatic hydrolysis temperature remained constant. At the same ethanol concentration, increasing the compound enzyme dosage first increased total anthocyanin yield from purple sweet potato, followed by a decrease after reaching the maximum. Similarly, an increase in ethanol concentration at the same compound enzyme dosage levels initially increased the yield, followed by a decline after reaching the maximum. The interaction between ultrasound temperature and compound enzyme dosage for total anthocyanin yield at constant ethanol concentration and enzymatic hydrolysis temperature (Figure 2B) also showed significance (*p* < 0.01). At constant compound enzyme dosage, elevating ultrasound temperature firstly increased total anthocyanin yield, which was later reduced. Finally, the interaction of compound enzyme dosage and enzymatic hydrolysis temperature (Figure 2C) also showed significance (*p* < 0.05). At constant enzymatic hydrolysis temperature, elevating compound enzyme dosage first increased total anthocyanin yield, which was later reduced.

### 2.5. Model Optimization and Verification

The extraction conditions optimized with Design Expert 8.0.6 were as follows: ethanol concentration 78% (predicted value, 77.1%), ultrasound temperature 48 °C (predicted value, 47.70 °C), compound enzyme dosage 1.3% (predicted value, 1.28%), and enzymatic hydrolysis temperature 41 °C (predicted value, 40.73 °C). Three parallel assays were carried out under the optimal conditions, yielding a total anthocyanin yield of 2.27 mg/g. The above findings indicate the BBD succeeded in optimizing anthocyanin extraction from purple sweet potato, with a precise and reliable prediction.

The yields of total anthocyanin extracted by UAEE were greater than those obtained using conventional extraction (CE) [29], ultrasound-assisted extraction (UAE) [29,30], or accelerated-solvent extraction [29]. UAEE may cause substantial cell wall breakdown, which adds to anthocyanin migration into the solvent. Furthermore, it might be attributed to ultrasonic and enzyme facilitating solvent penetration into the samples, resulting in increased anthocyanin solubility in the solvent. Nevertheless, the present results confirmed that UAEE is a suitable and valid method for the extraction of anthocyanins from PSP9.

### 2.6. Antioxidant Activities of the Extracted Anthocyanins

DPPH and hydroxyl radical scavenging assays are broadly utilized for evaluating the free radical-scavenging activities of antioxidants, as a rapid and reliable technique [31,32]. In general, the sample level needed to scavenge 50% of the tested radical (IC_50_) is a measure of antioxidant efficiency. Vitamin C (Vc) has a strong antioxidant activity, and is commonly used as a positive control [33]. The antioxidant features of anthocyanins from purple sweet potato which were extracted by the UAEE technique, remain undefined. Hence, DPPH and hydroxyl radical scavenging assays were carried out to examine anthocyanins extracted from purple sweet potato.

**Figure 3:**
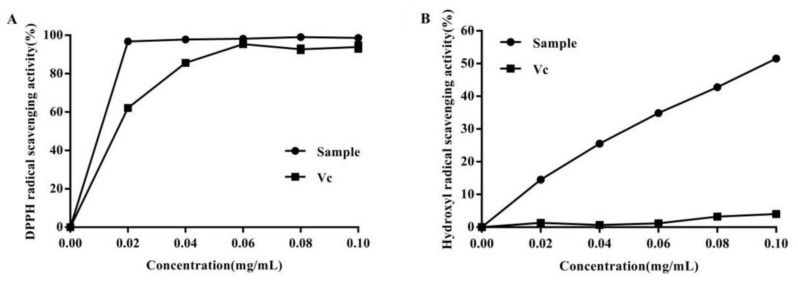
Antioxidant activity of total anthocyanins extracts. (**A**) DPPH radical scavenging activity. (**B**) Hydroxyl radical scavenging activity. Sample, total anthocyanins from purple sweet potato. Vc was used as a positive control.

Studies have shown that the DPPH radical can release a chromophore while receiving an electron from any hydrogen donor, and it is usually employed to detect the stability of a sample and antioxidant activity in the isolation process [34]. DPPH radical scavenging activity is widely utilized for the determination of the antioxidant features of vegetables, fruits, and food extracts. As shown in Figure 3A, total anthocyanins from purple sweet potato had high DPPH radical scavenging activity. At anthocyanin levels of 0.1 mg/mL, DPPH radical scavenging activity reached a maximum value of 99%, which was 1.05 times higher than that of Vc. This DPPH radical scavenging activity was concentration-dependent, increasing with the concentration of the extract. These findings suggest total anthocyanins from purple sweet potato have strong scavenging activity, with an IC_50_ of 0.089 μg/mL. The anthocyanins extracted by UAEE showed higher DPPH radical scavenging activity than those extracted under ultrasound-assisted extraction of total anthocyanins from *Rubia sylvatica* Nakai fruit [35]. Thus, the ultrasound-assisted compound enzymatic anthocyanin extract showed excellent antioxidant activity. As a result, the ultrasound-assisted compound enzymatic anthocyanin extract demonstrated exceptional antioxidant activity.

Similarly, dose dependency was also observed with hydroxyl radical scavenging capacity. The hydroxyl radical that possesses a strong reactivity can induce severe cell damage and is usually employed to determine the defense mechanism of a living body against antioxidants. The hydroxyl radical scavenging activities of total anthocyanins from purple sweet potato at different concentrations are shown in Figure 3B. At total anthocyanin levels of 0.1 mg/mL, hydroxyl radical scavenging capacity reached a maximum value of 51.5%, which was 12.875 times greater than that of Vc. Therefore, anthocyanins from purple sweet potato show strong hydroxyl radical scavenging capacity, with an IC_50_ of 100.229 μg/mL.

Previous studies have shown that anthocyanins produced by various plants have distinct antioxidant features [6,36]. It has been proposed that different hydroxylation, glycosylation, acylation, and methoxylation patterns of anthocyanins result in different antioxidant powers [37,38]. For example, the aglycone structure and the attached sugar moiety could scavenge O^2−^ and ONOO^−^ radicals. Therefore, the composition of specific anthocyanins, and their derivatives, from various colored plants might correlate with differences in antioxidant capacity. Hence, the UAEE method may contribute to protecting the structure of anthocyanins extracted from purple sweet potato and retaining of their antioxidant features. The extract is expected to be a natural antioxidant and a natural colorant in the food industry.

### 2.7. Identification of Anthocyanins by UPLC-MS

Individual anthocyanins from PSP9 extractions were identified using UPLC-MS (Figure S1, Supplementary Materials). UPLC chromatography confirmed the presence of five major anthocyanins in the final extracts, as shown in Table S1, with retention times of 12.08, 13.10, 13.30, 16.80, and 19.35 min for peaks 1–5. They were identified as Malvidin-acetaldehyde, Peonidin-3-caffeoyl sophoro-side-5-glucoside, Peonidin, Malvidin and Cyanidin based on molecular ion [M]^+^ and MS^+^ (*m*/*z*). The major peaks in Table S1 were examined using the associated literature on anthocyanin structure [30,39]. The dominant anthocyanidins in PSP9 were cyanidin, peonidin, and malvidin, which differ from previous reports that cyanidin and peonidin were the main anthocyanidins in purple sweet potato [40]. Many factors can influence the content and composition of anthocyanins in plants, including edaphic factors, such as environmental factors (soil and climate), genotype, and crop year within the same variety. Hence, malvidin and malvidin-acetaldehyde in PHBB anthocyanins may therefore depend on the species and the region. Some of the peaks in Figure S1 were not studied since their relative intensity was substantially lower than that of the prominent peaks. Total anthocyanin extraction from PSP9 using UAEE technology has never been investigated before, and our current study, when compared to other extraction methods, showed promising results in terms of the number of anthocyanin compounds extracted and the extraction yield.

## 3. Materials and Methods

### 3.1. Plants

PSP9 samples were purchased from Mianyang Institute of Agriculture Science, Mianyang, China, with a moisture content of 69.8%. The samples were dried using vacuum sealing at 55 °C for 8 h. Dried potatoes were ground by a pulverizer (FK-A, Jiangsu Jintan Jincheng Guosheng Experimental Instrument Factory Co., Ltd., Changzhou, China) to yield a 0.4-mm particle powder, which was stored at −20 °C.

### 3.2. Ultrasound-Assisted Enzymatic Extraction of Total Anthocyanins

Purple sweet potato powder (2 g) was mixed with an extraction solvent containing enzymes, including cellulase (Hefei Bomei Biological Technology, Hefei, China; enzyme activity, 105 U/g), pectinase (Hefei Bomei Biological Technology; Hefei, China; enzyme activity, 105 U/g) and papain (Nanning Pang Bo Biological Engineering, Nanning, China; enzyme activity, 3.0 × 10^4^ U/g). The mixture was left to react, and the reaction was stopped by boiling for 5 min. Subsequently, the extraction was performed on an ultrasonic cleaner (KQ5200DB, Kunshan Chaoshen Instrument, Kunshan, China). The obtained extracts underwent centrifugation at 3500× *g* for 20 min at ambient (KDC-1044, Anhui Zhongke Zhongjia Scientific Instrument, Hefei, China) to yield anthocyanin extracts, which were stored at 4 °C until analysis.

### 3.3. Total Anthocyanin Level Assessment

Total anthocyanins were quantitated by the pH-differential technique [41,42]. In brief, purple sweet potato specimens were added to buffer solutions at pH 1 and pH 4.5, respectively, and optical densities at 520 and 700 nm were obtained on a UV-1750 spectrophotometer (Nanchang Jiedao Scientific Instrument, Nanchang, China). Total anthocyanin amounts were derived as cyanidin-3-*O*-glucoside levels, as shown in Equation (1) (Equation (3)):

Total anthocyanin content (mg/g) = (A × Mw × DF × 1000)/(ε × L × m)(3)

where A = (A_520_ − A_700_) pH 1.0 − (A_520_ − A_700_) pH 4.5; Mw represents cyanidin-3-*O*-glucoside’s molecular weight, i.e., 449.2 g/mol; DF and ε are the dilution factor and extinction coefficient (26,900 L/mol cm), respectively; L represents the optical path (1 cm); m is the mass of dry Mianzishu 9 sample (g); V represents the total volume (mL); 1000 reflects conversion from g to mg.

### 3.4. Single-Factor Experiments

The effects of various factors on total anthocyanin yield were examined by single-factor experiments. Initially, 0.2 g of purple sweet potato powder was extracted sequentially with 4.0 mL of 60% ethanol containing 1.5% enzyme mixture (1:1:1 of cellulose: pectinase: papain; 50 °C enzymatic hydrolysis temperature, 4.5 enzymatic hydrolysis pH, and 1.5 h enzymatic hydrolysis time), followed by two ultrasonication sessions of 30 min each at 50 °C (ultrasound temperature). The effects of ethanol concentration, material-to-liquid ratio, compound enzyme dosage, compound enzyme ratio (cellulase: pectinase: papain), enzymatic hydrolysis temperature and pH, enzymatic hydrolysis time, ultrasound temperature, ultrasound time, and number of ultrasonication sessions were assessed by changing one factor with the others remaining unchanged during each assay. Therefore, ethanol was examined at 60%, 70%, 80%, 90%, and 100% with other parameters remaining fixed. Similarly, the material-to-liquid ratio was varied from 1:5 to 1:25 (g/mL), the compound enzyme dosage from 0.5% to 2.5%, the compound enzyme ratio (cellulase: pectinase: papain) from 2:2:1 to 1:1:1, the enzymatic hydrolysis temperature from 30 °C to 70 °C, the enzymatic hydrolysis pH from 4.0 to 6.0, the enzymatic hydrolysis time from 0.5 h to 2.5 h, the ultrasound temperature from 30 °C to 70 °C, the ultrasound time from 10 min to 50 min, and the number of ultrasonication sessions from 1 to 3.

### 3.5. Plackett-Burman Design (PBD)

The PBD was utilized for assessing the effects of six parameters affecting total anthocyanin extraction in single-factor analysis, namely compound enzyme dosage (X_1_), enzymatic hydrolysis pH (X_2_), enzymatic hydrolysis temperature (X_3_), material-to-liquid ratio (X_4_), ethanol concentration (X_5_) and ultrasound temperature (X_6_). Twelve combinations of these factors (X_1_–X_6_) applied at low (−) or high (+) levels were examined (Table 4). Levels for various factors resulted from single-factor analysis (Table 5), based on total anthocyanin yield. Assays were repeated thrice, and averages were utilized for fitting the PBD, following the linear model (Equation (4)):

(4) $$Y=\beta_{0}+\sum \beta_{i}x_{i}$$

where Y is the response (total anthocyanin content), β_0_ is a constant, β_i_ is the linear regression coefficient and x_i_ is the level of the independent variable.

### 3.6. Box-Behnken Design (BBD)

To determine the associations of the four factors with the greatest effects, i.e., ethanol concentration (X_1_), ultrasound temperature (X_2_), compound enzyme dosage (X_3_) and enzymatic hydrolysis temperature (X_4_), with total anthocyanin yield, a four-factor (X_1_–X_4_) three-level (−1, 0, +1) BBD was utilized to assess the variable combinations (Table 6). Factor ranges were based on single-factor analysis and PBD data. Response data was fitted to the RSM model, as shown in Equation (5):

(5) $$Y=\beta_{0}+\overset{3}{\underset{i=1}{\sum }}\beta_{i}x_{i}+\overset{3}{\underset{i=1}{\sum }}\beta_{\mathrm{ii}}x_{\mathrm{ii}}^{2}+\overset{2}{\underset{i=1}{\sum }}\overset{3}{\underset{j=i+1}{\sum }}\beta_{\mathrm{ij}}x_{i}x_{j}$$

where Y represents the response factor (total anthocyanin yield), X_i_ and X_j_ constitute independent factors, and β_0_, β_i_, β_ii_ and β_ij_ are constant, linear, quadratic, and cross-products coefficients, respectively.

### 3.7. Antioxidant Activity Assessment

#### 3.7.1. DPPH Radical Scavenging Assay

Experiments were conducted as previously reported by Yuan and colleagues [43], with slight modifications. Briefly, 2 mL of a serially diluted sample or ethanol (control) were mixed with the DPPH radical in ethanol (2 mL) (Shanghai Ika Biotechnology, Shanghai, China). The mixture was incubated at ambient for 30 min with vigorous shaking, shielded from light. The optical densities of the sample (OD_sample_) and blank (OD_blank_) were obtained at 517 nm on a spectrophotometer (Nanchang Jiedao Scientific Instrument, Nanchang, China). Vitamin C (Vc) served as a positive control. Parallel measurements were made thrice, and the mean value was calculated. The DPPH scavenging activity was derived as shown in Equation (6):

DPPH (%) = (1 − OD_sample_/OD_blank_) × 100 (6)

where OD_sample_ and OD_blank_ are optical densities of the sample and blank (ethanol), respectively. Regression analysis of serially diluted extracts was performed to determine IC_50_ values.

#### 3.7.2. Hydroxyl Radical Scavenging Capacity

The hydroxyl radical scavenging capacity of total anthocyanins from purple sweet potato was determined as described by Mau et al. [44], with Vc constituting a positive control. Parallel measurements were made thrice, and the mean value was calculated. Regression analysis of serially diluted extracts was performed to determine IC_50_ values.

### 3.8. Purifcation of Anthocyanins

The crude anthocyanins present in PSP9 were loaded onto a column (2.0 cm × 30 cm) of D101: AB-8 = 1:8 composite macro-porous resin. The column was washed with 2 L of deionized water at a flow rate of 1.5 mL/min to remove the majority of proteins, sugars, organic acids, and ions. The purple sweet potato anthocyanin loading concentration was 0.105 mg/mL, the pH of adsorption loading was 3, the flow rate of adsorption loading was 1.5 mL/min, the concentration of desorption ethanol was 60%, and anthocyanins were eluted using 75.5 mL of ethanol, 60% (*v*/*v*) ethanol at 1.5 mL/min. To obtain the purified anthocyanin samples, the eluate was concentrated with a rotary evaporator at 45 °C and freeze-dried.

### 3.9. Identification of Anthocyanins by HPLC-MS

The anthocyanins present in PSP9 were identified by Liquid Chromatography coupled to a triple stage quadrupole (TSQ) Quantum Ultra Mass Spectrometer (Thermo Fisher Scientific, Waltham, MA, USA). A hypersil gold column (150 mm × 2.1 mm, 5 m, Thermo) was used. The mobile phase consisted of 0.1% (*v*/*v*) formic acid (A) and CH_3_OH (B). The gradient elution program was as follows: 95% A at 0–5 min, 5–8 min, 95–80% A; 8–13 min, 80–40% A; 13–18 min, 40–5% A, 18–23 min, 5% A; and back to 95% A at 23.1 min, re-conditioning of the column for 7 min. The flow rate was 0.2 mL/min. The solution was ionized by means of an electrospray source in positive ionization mode with a desolvation gas flow of 600 L/h at 350 °C and a capillary cone voltage of 3000 V. The full-scan mode was applied in the range of 100–2000 *m*/*z*.

### 3.10. Statistical Analysis

The data were the mean and standard deviation (SD) of three independent experiments. One-way analysis of variance (ANOVA) with a Tukey multiple comparison test was performed for comparisons. The statistical significance was indicated by *p* < 0.05. SPSS v.24.0 (SPSS, Chicago, IL, USA) was utilized, as well as the Response surface of Design-Expert 8.0.6 Trial (Stat-Ease Inc., Minneapolis, MN, USA).

## 4. Conclusions

The UAEE method is rapid and effective for extracting total anthocyanins from purple sweet potato (Mianzishu No. 9). The optimal extraction conditions were 78% ethanol concentration, 1:15 (g/mL) material-to-liquid ratio, 2:2:1 enzyme ratio (cellulase: pectinase: papain), 1.3% compound enzyme dosage, 41 °C enzymatic hydrolysis temperature, pH 4.5 for enzymatic hydrolysis, 1.5 h enzymatic hydrolysis time, 48 °C ultrasound temperature, 20 min ultrasound time, and two ultrasonication sessions. The optimal yield was 2.27 mg/g. Importantly, the extract obtained by ultrasound-assisted enzymatic extraction was superior to Vc, pertaining to antioxidant function. A total of five anthocyanins were identified and characterized using UPLC-MS, which significantly added value to the existing knowledge of its nutraceutical composition.

The promising findings of this study should serve as a springboard for future research in a wide range of fields. For example, the UAEE technique could be used in the industrial extraction of anthocyanins to form enriched complexes or extracts that can be used in the food, pharmaceutical, or cosmetic industries. Furthermore, these findings inspire further research into the possible relationship between anthocyanin bioactivity and bioavailability, as well as the study of the stability, antioxidant activity, and bio-accessibility of anthocyanins from purple sweet potato (Mianzishu No. 9) using an in vitro simulated digestion.

## Figures and Tables

**Table 1:** Analysis of variance and regression analysis of PBD data for the prediction of significant extraction variables ^a^.

Scheme	Sum of Squares	df	Mean Squares	F-Value	Regression Coefficient	*p*-Value
Model ^b^	0.16	6	0.027	28.21	1.96	0.0011
X_1_	0.035	1	0.035	37.13	−0.054	0.0017 **
X_2_	0.0002	1	0.0002	0.21	0.00408	0.6664
X_3_	0.0095	1	0.0095	9.94	0.028	0.0253 *
X_4_	0.00017	1	0.00017	0.18	0.00379	0.6885
X_5_	0.11	1	0.11	111.94	−0.19	0.0001 **
X_6_	0.0094	1	0.0094	9.85	0.028	0.0257 *
Residual	0.00477	5	0.000955			
Pure Error	0.17	11				

^a^ Results were obtained using Design-Expert 8.0.6 software. ^b^ X_1_, compound enzyme dosage; X_2_, enzymatic hydrolysis pH; X_3_, enzymatic hydrolysis temperature; X_4_, material-to-liquid ratio; X_5_, ethanol concentration; X_6_, ultrasound temperature. Level of significance: ** Significant at *p* < 0.01, * Significant at *p* < 0.05.

**Table 2:** Anthocyanin extraction yield obtained from Box-Behnken design (BBD).

Run	Extraction Conditions				Total Anthocyanins/mg/g
	X_1_	X_2_	X_3_	X_4_	
1	0	−1	0	−1	2.17 ± 0.03
2	1	1	0	0	1.97 ± 0.02
3	0	1	−1	0	2.24 ± 0.02
4	−1	0	1	0	2.16 ± 0.02
5	−1	−1	0	0	2.06 ± 0.04
6	0	1	0	−1	2.05 ± 0.03
7	0	0	−1	−1	2.15 ± 0.03
8	0	0	−1	1	2.05 ± 0.08
9	0	0	0	0	2.35 ± 0.03
10	0	0	−1	1	2.03 ± 0.04
11	1	0	1	0	1.89 ± 0.10
12	0	1	1	0	2.20 ± 0.03
13	0	0	0	0	2.30 ± 0.02
14	0	0	0	0	2.25 ± 0.02
15	1	0	−1	0	2.07 ± 0.05
16	1	0	0	−1	1.90 ± 0.03
17	0	−1	0	1	2.05 ± 0.03
18	1	−1	0	0	2.00 ± 0.05
19	−1	1	0	0	2.16 ± 0.02
20	0	−1	1	0	2.32 ± 0.02
21	0	0	1	1	2.18 ± 0.05
22	1	0	0	1	1.95 ± 0.08
23	−1	0	−1	0	2.01 ± 0.06
24	−1	0	0	−1	2.03 ± 0.06
25	−1	0	0	1	2.00 ± 0.03
26	0	0	0	0	2.30 ± 0.03
27	0	−1	−1	0	1.99 ± 0.07
28	0	0	0	0	2.30 ± 0.03
29	0	1	0	1	2.13 ± 0.04

X_1_, ethanol concentration; X_2_, ultrasound temperature; X_3_, compound enzyme dosage; X_4_, enzymatic hydrolysis temperature.

**Table 3:** Analysis of Variance (ANOVA) for response surface polynomial model.

Source	Sum of Squares	df	Mean Squares	F-Value	*p*-Value
Model	0.45	14	0.032	16.93	<0.0001
X_1_	0.033	1	0.033	17.50	0.0009 **
X_2_	0.002061	1	0.002061	1.08	0.3167
X_3_	0.005889	1	0.005889	3.08	0.1011
X_4_	0.00006225	1	0.00006225	0.033	0.88594
X_1_ X_2_	0.004239	1	0.0004239	2.22	0.1587
X_1_ X_3_	0.028	1	0.028	14.82	0.0018 **
X_1_ X_4_	0.00167	1	0.00167	0.87	0.3659
X_2_ X_3_	0.032	1	0.032	16.97	0.0010 **
X_2_ X_4_	0.009034	1	0.009034	4.72	0.0474
X_3_ X_4_	0.015	1	0.017	7.83	0.0143 *
X_1_^2^	0.25	1	0.25	132.09	<0.0001 **
X_2_^2^	0.020	1	0.020	10.54	0.0055 **
X_3_^2^	0.024	1	0.024	12.39	0.0034 **
X_4_^2^	0.12	1	0.12	64.47	<0.0001 **
Residual	0.027	14	0.001912		
Lack of Fit	0.022	10	0.002194	1.82	0.2964
Pure Error	0.004827	4	0.001207		
Sum	0.48	28			

X_1_, ethanol concentration; X_2_, ultrasound temperature; X_3_, compound enzyme dosage; X_4_, enzymatic hydrolysis temperature; df represent degree of freedom. Level of significance: ** Significant at *p* < 0.01, * Significant at *p* < 0.05.

**Table 4:** Variables and levels encoded for PBD.

Input Variables	Levels	
	−1	1
compound enzyme dosage (%) (X_1_)	0.5	1.5
enzymatic hydrolysis pH (X_2_)	4	5
enzymatic hydrolysis temperature (°C) (X_3_)	30	50
material-to-liquid ratio (g/mL) (X_4_)	1:15	1:25
ethanol concentration (%) (X_5_)	80	90
ultrasound temperature (°C) (X_6_)	40	60

**Table 5:** Anthocyanins extraction yield obtained from PBD.

Runs	X_1_	X_2_	X_3_	X_4_	X_5_	X_6_	Total Anthocyanins/mg/g
1	−1	1	−1	1	−1	1	2.25 ± 0.04
2	1	−1	1	−1	1	1	1.97 ± 0.04
3	1	−1	−1	1	−1	1	2.10 ± 0.04
4	−1	−1	1	1	1	1	2.07 ± 0.03
5	−1	−1	−1	−1	−1	−1	2.13 ± 0.06
6	1	1	−1	−1	1	1	1.90 ± 0.08
7	−1	−1	−1	−1	1	−1	1.97 ± 0.02
8	1	1	−1	1	1	−1	1.84 ± 0.03
9	−1	1	1	1	1	−1	2.03 ± 0.04
10	1	−1	1	1	−1	−1	2.09 ± 0.04
11	−1	1	1	−1	−1	1	2.23 ± 0.05
12	1	1	1	−1	−1	−1	2.13 ± 0.04

X_1_, compound enzyme dosage; X_2_, enzymatic hydrolysis pH; X_3_, enzymatic hydrolysis temperature; X_4_, material-to-liquid ratio; X_5_, ethanol concentration; X_6_, ultrasound temperature.

**Table 6:** Experimental values and coded levels of the independent variables used for the Box-Behnken design (BBD).

Factor Levels	Independent Variable			
	X_1_ (%)	X_2_ (°C)	X_3_ (%)	X_4_ (°C)
−1	70	40	0.5	30
0	80	50	1.0	40
+1	90	60	1.5	50

X_1_, ethanol concentration; X_2_, ultrasound temperature; X_3_, compound enzyme dosage; X_4_, enzymatic hydrolysis temperature.

## Data Availability

The data presented in this work are available in the article.

## Supplementary Materials

The following supporting information can be downloaded at: https://www.mdpi.com/article/10.3390/molecules27144344/s1, Figure S1: UPLC-MS of anthocyanins from the purple sweet potato (Mianzishu 9) extractions; Table S1: Characterisation of anthocyanins in purple sweet potato (Mianzishu 9) using UPLC-MS.

## Sample Availability

Samples of the compounds are not available from the authors.
