# Supplementary material for: Extracting Total Anthocyanin from Purple Sweet Potato Using an Effective Ultrasound-Assisted Compound Enzymatic Extraction Technology
Source: Molecules. 2022 Jul 6;27(14):4344. doi: 10.3390/molecules27144344 (PMC9317032; doi:10.3390/molecules27144344)
Supplement: Supplementary file 1 [file molecules-27-04344-s001.zip › molecules-1788979-supplementary.pdf]

molecules

MDPI

Supplementary Materials

Extracting Total Anthocyanin from Purple Sweet Potato Using an Effective Ultrasound-Assisted Compound Enzymatic Extraction Technology

Fang Wang [1], Shuo Zhang [1], Guowei Deng [1,*], Kun Xu [1], Haiyan Xu [2] and Jialei Liu [3,*]

[1] Sichuan Provincial Key Laboratory for Development and Utilization of Characteristic Horticultural Biological Resources, College of Chemistry and Life Sciences, Chengdu Normal University, Chengdu 611130, China; wangfangbia@163.com (F.W.); 15388114168@163.com (S.Z.); 091044@cdnu.edu.cn (K.X.)
[2] College of Life Sciences, Sichuan Normal University, Chengdu 610101, China; weiliangxhy@163.com
[3] Institute of Environment and Sustainable Development in Agriculture, Chinese Academy of Agricultural Sciences, Beijing 100081, China
* Correspondence: guoweideng@cdnu.edu.cn (G.D.); liujialei@mail.ipc.ac.cn (J.L.)

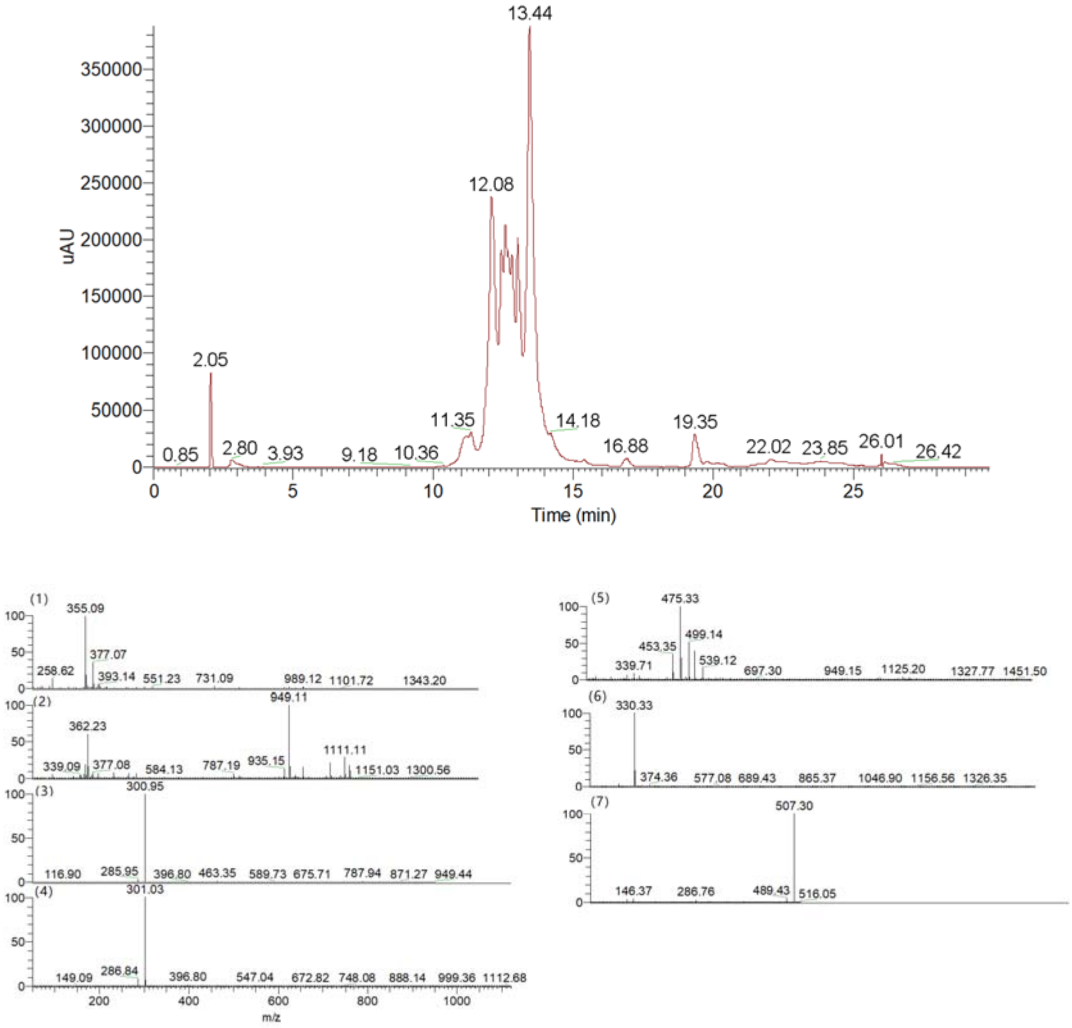

Figure S1. UPLC-MS of anthocyanins from the purple sweet potato (Mianzishu 9) extractions.

Table S1. Characterisation of anthocyanins in purple sweet potato (Mianzishu 9) using UPLC-MS.

Peak	RT (time)	M+ (*m*/*z*)	MS+ (*m*/*z*)	Compound	References
1	12.08	355		Malvidin-acetalde-hyde	[1]
2	12.65	1111	949, 787,362	Unknown 1	
3	13.10	949	787,463,301	Peonidin-3-caffeoyl sophoroside-5-glu-coside	[2]
4	13.30	301.03	-	Peonidin	[1]
5	13.44	1125	949,475	Unknown 2	
6	16.80	330.33	-	Malvidin	[1]
7	19.35	507	287,146	Cyanidin	[1]

References

1. Wang, H.B.; Race, E.J.; Shrikhande, A.J. Characterization of anthocyanins in grape juices by ion trap liquid chromatography-mass spectrometry. *J. Agric. Food Chem.* **2003**, *51*, 1839–1844.
2. Zhu, Z.; Guan, Q.; Koubaa, M.; Barba, F.J.; Roohinejad, S.; Cravotto, G.; Yang, X.; Li, S.; He, J. HPLC-DAD-ESI-MS^2 analytical profile of extracts obtained from purple sweet potato after green ultrasound-assisted extraction. *Food Chem.* **2017**, *215*, 391–400.
